# Passive exposure to speech sounds induces long-term memory representations in the auditory cortex of adult rats

**DOI:** 10.1038/srep38904

**Published:** 2016-12-20

**Authors:** Jari L. O. Kurkela, Arto Lipponen, Jarmo A. Hämäläinen, Risto Näätänen, Piia Astikainen

**Affiliations:** 1 Department of Psychology, University of Jyväskylä, Jyväskylä, Finland; 2 Institute of Psychology, University of Tartu, Tartu, Estonia; 3 Center of Functionally Integrative Neurosciences (CFIN), University of Århus, Århus, Denmark; 4 Cognitive Brain Research Unit, Institute of Behavioural Sciences, University of Helsinki, Helsinki, Finland

## Abstract

Experience-induced changes in the functioning of the auditory cortex are prominent in early life, especially during a critical period. Although auditory perceptual learning takes place automatically during this critical period, it is thought to require active training in later life. Previous studies demonstrated rapid changes in single-cell responses of anesthetized adult animals while exposed to sounds presented in a statistical learning paradigm. However, whether passive exposure to sounds can form long-term memory representations remains to be demonstrated. To investigate this issue, we first exposed adult rats to human speech sounds for 3 consecutive days, 12 h/d. Two groups of rats exposed to either spectrotemporal or tonal changes in speech sounds served as controls for each other. Then, electrophysiological brain responses from the auditory cortex were recorded to the same stimuli. In both the exposure and test phase statistical learning paradigm, was applied. The exposure effect was found for the spectrotemporal sounds, but not for the tonal sounds. Only the animals exposed to spectrotemporal sounds differentiated subtle changes in these stimuli as indexed by the mismatch negativity response. The results point to the occurrence of long-term memory traces for the speech sounds due to passive exposure in adult animals.

The cerebral cortex is highly adaptive to the sensory environment, and the acoustic environment has a strong effect on cortical representations of sounds in animals^1^,^2^ and humans^3^,^4^,^5^, especially within a critical period during early development. In human infants, auditory perceptual learning, including learning of speech sound discrimination, occurs automatically without direct attention, even during nocturnal sleep^3^.

After the critical period, auditory perceptual learning in humans is thought to be efficient only when supported by attentive training^4^,^5^. There is also a critical period for auditory learning in young animals^6^,^7^, after which behaviourally relevant stimuli are required for perceptual learning (for a review, see^6^). The application of behaviourally meaningful stimuli in adult animals has been shown to induce learning-related plastic changes in cortical maps in response to different types of stimulation, such as pure tone frequencies^8^,^9^, sound intensities^10^, temporal modulation rates^11^, and speech sounds^12^.

In humans, cortical map changes have seldom been investigated because this would involve the use of an invasive method. However, electrophysiological recordings of so-called mismatch negativity (MMN)^13^ (for a review, see^14^) can be done non-invasively, and these revealed perceptual learning-related plasticity in cortical sound representations of adult humans due to active training^15^,^16^,^17^. In those studies, a few hours of training, usually for 3–6 d, caused long-term memory traces for the trained sounds.

A few studies have examined the effects of passive exposure on animals and humans. Studies of passive sound exposure in animals focused on the formation of sound representations (cortical maps) in the auditory cortex and did not detect observable effects, even after several days of exposure^1^,^6^,^11^. In a human study, passive listening to vowels for approximately of one h did not improve sound discrimination ability or produce any changes in ERPs^18^.

In anaesthetized animals, MMN, known also as the mismatch response (MMR), in local-field potentials (LFPs) and stimulus-specific adaptation (SSA) in single-cell responses have been observed, pointing to rapid perceptual learning within a single recording session (for reviews, see^19^,^20^,^21^,^22^). Modulation of MMN^23^,^24^,^25^,^26^ and SSA^27^,^28^,^29^ in response to changes in pure tone frequency, syllables, and other complex stimuli are reported in the auditory cortex, as well as in subcortical structures in the auditory pathway (MMN^30^ and SSA^31^,^32^, for a review, see^33^).

The aforementioned findings on MMN and SSA in animals are intriguing, as they demonstrate learning-related functional changes in the brain activity of adult animals, in the absence of behavioural relevance of the stimuli. Thus far, these responses have been shown only during a single session of passive sound exposure, indicating that short-term memory representations of the sounds are sufficient for their elicitation. It is not known, however, if the effect of passive exposure can be long-lasting, leading to formation of long-term memory representations.

To test whether the passive exposure can form long-term memory representations, we first exposed adult rats to behaviourally irrelevant speech sounds for 36 h on three consecutive nights for 12 h/d. We applied a statistical learning paradigm, i.e. an oddball condition, with a large number of stimuli presented to the animals (total of approximately 250,000 sounds). Since the exposure was delivered during three consecutive nights, the consolidation of memory traces for speech sounds was expected to occur^15^,^16^. In a between-group design, two groups of freely moving animals were exposed to two different continuous sequences of speech sounds. For one group, the sequence consisted of repetitions of a vowel/a/spoken by a Finnish speaker with changes in its spectrotemporal features. For the other group, tonal changes were presented in repetitions of a vowel/a/spoken by a Chinese speaker. Both the standard and deviant stimuli in the sound series were different between the animal groups, allowing the groups to serve as controls for each other. The sounds were presented in the oddball condition, in which a rarely presented deviant sound was occasionally interspersed with a standard sound, with an inter-stimulus interval (offset to onset) of 335 ms. Two different deviant sounds were interspersed with standard sounds: one with a large and the other with a small physical difference from the standard.

To reveal possible long-term memory traces for the speech sounds, LFPs in the auditory cortex of anesthetized rats were measured after the exposure ended. The same sound series that were applied in the exposure phase were also used in the test phase, but both the spectrotemporal and tonal changes were now presented to both animal groups, allowing comparison of the naive and exposed animals’ responses. In humans, MMN is a reliable indicator of auditory discrimination ability (for a review, see^14^), including speech sound discrimination ability^15^,^34^. An increase in the MMN amplitude^15^,^17^ and a decrease in latency^35^,^36^ reflects enhanced sound discrimination ability. Therefore, in the present study, we expected to observe increased MMN amplitudes and decreased latencies in response to the specific type of sound exposure.

## Results

**Figure 1:**
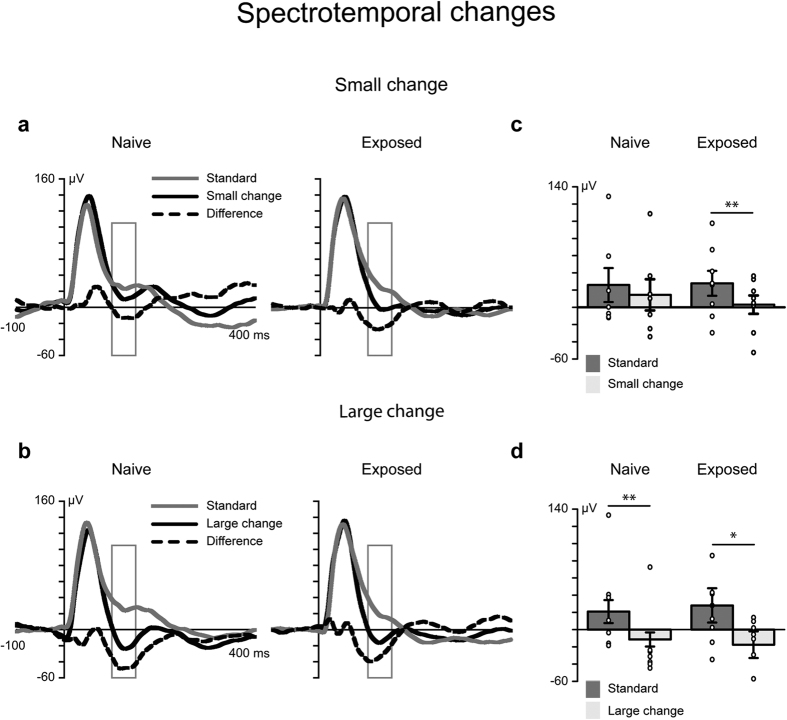
Passive exposure to spectrotemporal changes enhanced cortical responses to the same sounds presented later. **(a,b)** The grey line denotes responses to frequently presented standard sounds, and the black line denotes the responses to large or small changes in sound (deviant). The black dashed line represents the differential response (MMN), which was calculated by subtracting the response to the standard sound from the response to the deviant sound. The rectangles show the region of interest (mean amplitude values 100–150 ms after the stimulus onset). A repeated measures analysis of variance (ANOVA) of the factors ‘stimulus’ (standard vs. deviant), ‘deviant type’ (small vs. large change) and ‘group’ (exposed, *n* = 8 vs. naive, *n* = 7) revealed a three-way interaction effect: F_1, 13_ = 4.85, *P* = 0.046, η^2^_p_ = 0.272. (**c,d**) Response amplitudes for each animal (marked with circles) and the error bars indicating the mean and standard error of the mean values, *P < 0.05, **P < 0.01. (**c**) The small change in spectrotemporal features elicited the MMN response (M = −32.36 μV, SEM = ±10.49) in the exposed animals (*t*[7] = 3.08, *P* < 0.009, *d* = 1.258) but not in the naive animals (M = −11.28 μV, SEM = ±9.16) (*t*[6] = 1.232, *P* = 0.264, *d* = 0.465). (**d**) The large change in spectrotemporal features elicited the MMN in both the naive (M = −45.61 μV, SEM = ±9.80) and exposed animal (M = −32.36 μV, SEM = ±10.49) groups (*t*[6] = 4.66, *P* < 0.003, *d* = 1.760 and *t*[7] = 3.08 *P* < 0.018, *d* = 1.090, respectively).

**Figure 2:**
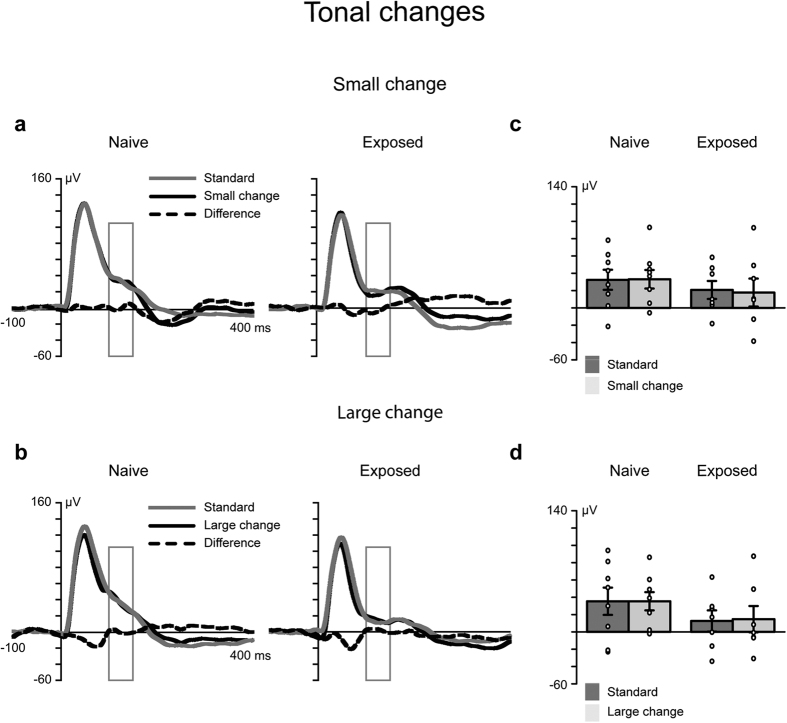
Tonal changes elicited neither an MMR response nor an exposure effect. **(a,b)** The grey line denotes the responses to frequently presented standard sounds, and the black line represents the responses to large or small changes in the deviating sound. The black dashed line represents the differential response (MMR), which was calculated by subtracting the response to the standard sound from the response to the deviant sound. The rectangles show the region of interest (mean amplitude values 100–150 ms after the stimulus onset). A repeated measures analysis of variance (ANOVA) of the factors ‘stimulus type’ (standard vs. deviant), ‘deviant type’ (small vs. large change) and ‘group’ (exposed, *n* = 7 vs. naive, *n* = 8) revealed no statistically significant effects. (**c,d**) Response amplitudes for each animal (marked with circles) and the error bars indicating the mean and standard error of the mean values.

All the stimuli elicited an auditory response, which peaked approximately 50 ms after the stimulus onset (Figs 1 and 2). Analyses of the mean amplitude values 100–150 ms after the onset of the stimulus was chosen a priori, based on earlier studies of MMN latency in rats^37^,^38^ and grand-averaged waveforms. The ANOVA and post-hoc tests revealed that large spectrotemporal changes elicited MMN in both animal groups, whereas small spectrotemporal changes elicited MMN only in the group of animals exposed to these stimuli (for details, see Fig. 1). Tonal changes did not elicit MMN in either animal group, and no exposure effect was observed (Fig. 2).

To determine the effect of the sound exposure on neural discrimination ability we compared the differential responses (the deviant minus the standard) by applying time-point-by-time-point t-tests (permutation tests) not only on the MMN latency but also on the whole waveform. First, the differential response to both spectrotemporal and tonal changes was compared against zero within each animal group separately. Second, if both animal groups showed a statistically significant differential response, the differential responses of the groups were compared to determine possible differences in latency.

With regard to the spectrotemporal sounds, large changes elicited a statistically significant differential response (MMN) in the exposure group 91–119 ms after the onset of the stimulus (smallest *P* = 0.034, 95% CI [−62.82–(−18.50)]; largest significant *P* = 0.049, 95% CI [−53.50–(−2.91)]). In the animal group naive to these sounds, MMN occurred 86–170 ms after the stimulus onset (smallest *P* = 0.003, 95% CI [−64.37–(−25.96)]; largest significant *P* = 0.049, 95% CI [−32.06–(−5.32)]). The between-group comparisons of the differential responses (deviant vs. standard) revealed no differences.

The small spectrotemporal changes elicited differential responses only in the group exposed to these stimuli. A significant differential response was observed 90–156 ms after the onset of the stimulus (smallest *P* = 0.003, 95% CI [−64.37–(−25.96)]; largest *P* = 0.049, 95% CI [−32.06–(−5.32)]).

With regard to the tonal changes, differential responses were not found in either group.

## Discussion

Herein, by applying an electrophysiological method, we demonstrated that passive auditory exposure can induce plastic changes in the function of the auditory cortex, even in adult animals. Passive exposure to speech sound changes for 3 d for a total of 36 h was sufficient to induce long-term memory representations of the sounds. This was observed as emergence of the MMN response for the small changes in spectrotemporal sounds in the animal group exposed to these sounds but not in the group of animals exposed to different sounds.

Previous studies failed to show effects of passive exposure on reorganization of the cortical maps of the auditory cortex in adult animals^1^,^11^,^39^. It is not clear whether the positive finding in the present study of the effectiveness of passive exposure on auditory cortex plasticity is related to the type of measurement (LFPs vs. single-cell responses), stimulus condition (oddball condition vs. repetition of one stimulus) or other methodological differences between the present and previous experiments.

We used LFPs to measure the co-activity of local neural networks, whereas previous studies employed single-cell responses^1^,^11^,^39^. LFPs capture synchronized synaptic potentials, afterpotentials of somatodendritic spikes and voltage-gated membrane oscillations^40^,^41^,^42^. As a result, LFPs are sensitive to sub-threshold neural processes and carry information about the state of the neural networks.

Another novel aspect of the present study was the use of a statistical learning paradigm (i.e. oddball condition), both in the exposure and test phases, in which rare changes in sounds are presented. Changes in stimulus environment can signal threat to animal and their biological significance is thus high. Previous studies applied only one stimulus type (pure tone^1^,^35^ or temporal modulation rates in pure tones^11^).

Previous studies that failed to show an effect of passive exposure on cortical sound representations in adult animals used a longer exposure time than that employed in the present experiment. For example, De Villers-Sidani *et al*.^35^ exposed rats for 3 d, 24 h/d. In other studies, the exposure times were 19 d, 1–16 h/d^1^ and up to 2 mon, 2 h/d^11^. Thus, the exposure time in our study compared to that of previous studies seems not to explain the different findings.

The number of stimulus presentations during the exposure period could also be important. We used a short inter-stimulus interval between the stimuli (335 ms, offset-to onset). Thus, approximately two stimuli per second and more than 250,000 stimuli within 36 h were presented to the animals. The large amount of repetition of the stimuli, together with the statistical learning paradigm and neural network-level electrophysiological recording, may have enabled us to observe the effect of passive exposure.

A recent study by Kato *et al*.^43^ that utilized two-photon calcium imaging in mice demonstrated that passive exposure to simple tones for 5 d (20 min/d) caused a progressive increase in the number of cells (layer 2/3 pyramidal cells of A1) showing inhibition by the tone presentation. Our data cannot provide conclusive evidence on the neural mechanism of the observed perceptual learning. Since the state of the brain and also the level of anaesthesia fluctuate temporarily distant responses cannot be reliably compared. This prevented direct comparison of standard and deviant responses between animal groups and instead group differences were investigated by comparing the differential responses. However, the finding by Kato *et al*.^43^ is in line with that of studies of SSA^27^,^28^,^29^. In those studies, SSA occurred in response to standard sounds (habituation), enabling the detection of the deviant sounds. It can be speculated that in our present experiment, the formation of the memory trace to the standard stimulus during the exposure period enabled better deviance detection, as indexed by the emergence of MMN for small spectrotemporal change only in the exposure group but not that in the group of animals exposed to different sounds.

In the present study, only exposure to spectrotemporal, not tonal, changes was effective in modulating MMN. The absence of MMN to tonal changes in both the exposed and naive animal groups indicated that the rat brain did not differentiate between the tonal features. This finding was somewhat surprising, as MMN in rats is elicited by rising and falling sinusoidal tones^44^. It can be assumed that tonal changes that occur gradually (i.e. over a period of 200 ms) are more difficult for the rat brain to differentiate than fast changes in sound frequency.

As demonstrated by the response latency, the MMN was sensitive to subtle changes in spectrotemporal features of human speech sounds at the beginning of the sounds but not to decrements in the duration of these sounds (Fig. 1). The latency of the MMN in the present experiment was somewhat longer than that of the MMN to frequency changes in pure tones (60–100 ms post-stimulus^23^). This is in line with the idea that the MMN latency reflects the complexity of the underlying cognitive process^14^. Indeed, we observed even longer latency responses (217 ms after the onset of the change) in rats to changes in abstract rules in syllables^45^. However, in the present study, the response latency was not shorter in the exposure group than in the naive group for the large spectrotemporal changes which elicited the MMN response in both groups. Similarly in a previous study in humans, active training modulated amplitude, but not latency, of the MMN response^46^.

Future studies should investigate the time required for passive exposure to induce plastic changes. As we measured the neural discrimination ability after only 3 d of exposure, not for example, after each night, it is not possible to conclude whether a smaller amount of exposure would also induce plastic changes in the function of the auditory cortex.

In sum, we found that rats could detect subtle changes in spectrotemporal features of human speech sounds, as indicated by electrophysiological responses recorded from the auditory cortex. Further, after exposure for 36 h, the change detection response of the group passively exposed to these sounds was enhanced but not that of the group exposed to different sounds. Our findings thus demonstrate that passive exposure to speech sound changes can induce long-term memory representations.

## Methods

### Animals

The study consisted of 15 adult male Wistar rats aged 24.5 ± 1.8 wk weighing 490.5 ± 44.4 g (mean ± SD). The estimation of the number of animals required for the experiment was based on a previous rat study of the detection of changes in responses to speech sounds^45^. The animals were individually housed and maintained in a 12-h light/dark cycle (lights on at 7.00 a.m.). All the experimental procedures and animal care protocols were approved by the National Experiment Board in Finland (licence ESAVI/10646/04.10.07/2014) and were in accordance with the European Communities Council Directive (86/609/EEC) regarding the care and use of animals for experimental procedures. After the experiment, the animals first received an overdose of urethane, and they were then sacrificed by cervical dislocation.

### Exposure

The animals were randomly divided into two groups. One group was exposed to tonal changes (*n* = 7), and the other was exposed to spectrotemporal changes (*n* = 8). Both groups were exposed to the sounds for three consecutive nights (12 h/night from 7 p.m. to 7 a.m.), giving a total exposure time of 36 h. The animals were exposed to the sound stimuli in their home cages from a passive loudspeaker system (StudioPro 3, M-Audio Inc., Cumberland, USA), which was directed towards the cages. The sound pressure level for each tone was 70 dB, as measured using a sound-level meter (type 2235, Bruel & Kjaer, Nærum Denmark), with C-weighting (optimized for 40–100 dB measurement).

### Stimuli and procedure

**Figure 3:**
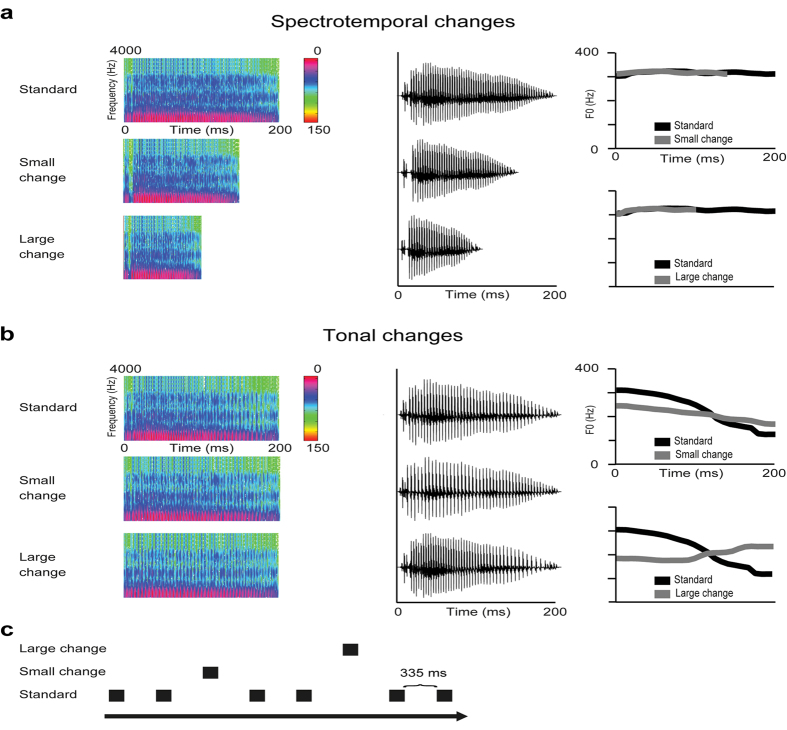
Sounds and the stimulus condition applied in the exposure and test phases. **(a,b)** Spectrograms, waveforms and fundamental frequencies of the sounds. The animals were exposed to either spectrotemporal (**a**) or tonal changes (**b)** in the vowel/a/. (**c**) The sounds were presented in an oddball series, where frequently presented standard sounds were interspersed with a large or small change in the sound. After the exposure, the LFPs in response to both stimulus types were measured in all the animals. The sounds were presented in the oddball condition, where frequently occurring standard stimuli (probability of 0.80) were interspersed with two deviant sounds (large or small change, probability of 0.10 each), using E-prime 1.2. software (Psychology Software Tools Inc., Sharpsburg, US). The inter-stimulus interval was 335 ms (offset to onset). The stimuli were delivered in a pseudorandom fashion, with the restriction that consecutive deviant sounds were separated by at least two standard sounds.

In the tonal stimulus series, the animals were exposed to different tones of the speech sound/a/. The sounds were prepared so that first phoneme/a/was spoken by a female native Chinese speaker with rising (i.e. Chinese lexical tone 2) and falling (i.e. Chinese lexical tone 4) lexical tones, and they were recorded at a sampling rate of 44.1 kHz. The sounds were then digitally edited using SoundForge software (SoundForge 9, Sony Corporation, Japan) to ensure they had a constant duration of 200 ms. To isolate the lexical tones and keep the rest of the acoustic features identical, pitch tier transfer was performed using Praat software (Praat v5.4.06, University of Amsterdam). Pitch tier transfer generated a rising tone and a falling tone, which were identical to each other, except for a pitch contour difference in fundamental frequency (F0). These two tones were taken as the endpoint stimuli to create a continuum of lexical tones with 10 interval steps. A morphing technique was performed in Matlab (The MathWorks, Inc., MA, US), with a STRAIGHT tool^47^ to create the three tones applied in the experiment. The repeatedly presented standard sound was the falling tone, and deviant sounds were a slightly falling tone (small change) and a rising tone (large change) (Fig. 3), corresponding to the tone continua 11, 7 and 3, respectively, as reported previously in detail elsewhere^48^. All the stimuli were normalized to have the same root mean square intensity.

In the spectrotemporal stimulus series, changes in the speech sound/a/were presented. They were prepared so, that phoneme/a/was first spoken by a female native Finnish speaker, and it was recorded at a sampling rate of 44.1 kHz. The sound was then digitally edited using SoundForge software (SoundForge 9, Sony Corporation, Japan) to ensure it had a constant duration of 200 ms. A morphing technique was performed in Matlab (The MathWorks, Inc., MA, US), using a STRAIGHT tool to modify the length of the stimuli and keep the fundamental frequency (F0) constant (Fig. 3). The repeatedly presented standard sound was the vowel/a/, which was 200 ms in duration. The deviant sounds were a 150-ms sound (small change) and 100-ms sound (large change) (Fig. 3). The method for shortening the sounds caused differences between the standard and deviant sounds immediately at the beginning of the sound.

### Surgery and LFP recording

After the exposure to the sounds, on the same day the exposure ended (2–7 h after the exposure), the animals were anesthetized with intraperitoneal injections (1.2 g/kg dose, 0.24 g/ml concentration) of urethane (Sigma-Aldrich, St. Louis, MO, US). Supplemental doses were injected if the required level of anaesthesia was not obtained. The level of anaesthesia was monitored by testing the withdrawal reflexes. The animals were rehydrated with a 2 ml saline injection (subcutaneous) every 2 h.

The head of the animal was attached to a stereotaxic instrument (David Kopf Instruments, Model 962, Tujunga, CA, US). Under local anaesthesia (lidocaine 20%, Orion Pharma, Espoo, Finland), the skin and underlying muscles were removed, and a unilateral craniotomy was performed to expose a 2 × 2 mm region of dura over the auditory cortex in the left hemisphere (4.5–6.5 mm posterior and 6–8 mm ventral to bregma). The tip of a PFA-insulated silver wire (A-M Systems, Chantilly, VA, US), 200 μm in diameter, was positioned on the surface of the dura. Although the coordinates for the craniotomy refer to the primary auditory cortex, some activity from the higher sensory areas may also have been captured. The latter is due to individual differences in the organization of the primary auditory cortex and the relatively large size of the tip of the electrode, allowing the signal to be conducted from adjacent areas.

A 29 G injection needle (Terumo, Leuven, Belgium) in the cerebellum served as the reference point, and a similar injection needle located under the neck skin provided the grounding point. Before recording the LFP, a headstage, composed of a screw and dental acrylic, was attached to the right prefrontal part of the skull to hold the head in place and allow removal of the right ear bar.

The auditory evoked potentials of all the animals to both the spectrotemporal and tonal changes were measured in separate blocks in a counterbalanced order between the animals. Both the spectrotemporal sound series and tonal sound series consisted of 1600 standard sounds and 400 deviant sounds (200 small and 200 large changes). The properties of the stimuli and presentation conditions were the same as in the exposure phase.

A continuous electrocorticogram was first 10-fold amplified using a low-noise MPA8I pre-amplifier (MultiChannel Systems MCS GmbH, Reutlingen, Germany). The signal was further fed to a filter amplifier (FA64I, filter: 1–5000 Hz, MCS). All the signals were digitized (USBME-64 System, MCS) and recorded with McRack software (MCS), using a 2000-Hz sampling rate. Finally, all the signals were digitally band-pass filtered between 1 and 500 Hz (high-pass: low-pass: fourth-order Bessel).

### Data analysis

The data were off-line filtered at 0.1–30 Hz (24 dB/octave). Sweeps from 100 ms before to 400 ms after the stimulus onset were averaged separately for both types of the deviant sounds and standard sounds that immediately preceded the deviant sounds. The averaged waveforms were baseline-corrected against the mean of a 100-ms pre-stimulus period.

### Statistics

The mean amplitude values from a time window of 100–150 ms from the stimulus onset were included in a repeated measures analysis of variance (ANOVA), with the within-subjects factors ‘stimulus type’ (standard vs. deviant) and ‘deviant type’ (small vs. large change) and a between-subjects factor ‘group’ (exposed vs. naive). Separate ANOVAs were performed for spectrotemporal and tonal sound series. The averaged data was normally distributed (Shapiro-Wilk test all P-values > 0.05) enabling to use parametric statistical tests.

Two-tailed one-sample *t*-tests were used as post-hoc tests to further investigate the interaction effects found in the ANOVA. Huynh–Feldt-corrected degrees of freedom were used whenever the sphericity assumption was violated. The corrected *P* values are reported, but the degrees of freedom are reported as uncorrected. Partial eta squared (η^2^_p_) was used as an index of the effect size estimates for the ANOVA, and Cohen’s *d* of that for the *t*-tests. *P* values smaller than 0.05 were considered significant.

We also compared the differential responses (deviant response minus the standard response) against zero (two-tailed one-sample *t-*test, permutation statistics as implemented in IBM Statistics, SPSS 24) at consecutive time points to determine the latency of a significant MMN response and detect possible differences in other than the MMN latency. To reveal only robust differences, 20 consecutive time points (corresponding to a 10-ms time segment) were required to show a significant difference. The *P*-values (*P* < 0.05) after bootstrapping with 1000 permutations are reported, together with their 95% confidence intervals.

## Additional Information

**How to cite this article**: Kurkela, J. L. O. *et al*. Passive exposure to speech sounds induces long-term memory representations in the auditory cortex of adult rats. *Sci. Rep.* **6**, 38904; doi: 10.1038/srep38904 (2016).

**Publisher's note:** Springer Nature remains neutral with regard to jurisdictional claims in published maps and institutional affiliations.
